# Supplementary material for: Role and mechanism of NCAPD3 in promoting malignant behaviors in gastric cancer
Source: Front Pharmacol. 2024 Apr 22;15:1341039. doi: 10.3389/fphar.2024.1341039 (PMC11070777; doi:10.3389/fphar.2024.1341039)
Supplement: Supplementary file 11 [file DataSheet2.ZIP › GSEA/Transcipt factor/my_analysis.Gsea.1599461814734/IRF5_TARGET_GENES.html]

Details for gene set IRF5\_TARGET\_GENES[GSEA]

|  || Dataset | filtered\_dataset.sample\_info.cls#WT\_versus\_NCAPD3\_MUT |
| Phenotype | sample\_info.cls#WT\_versus\_NCAPD3\_MUT |
| Upregulated in class | WT |
| GeneSet | IRF5\_TARGET\_GENES |
| Enrichment Score (ES) | 0.26823974 |
| Normalized Enrichment Score (NES) | 1.6626899 |
| Nominal p-value | 0.012461059 |
| FDR q-value | 0.54134434 |
| FWER p-Value | 1.0 |
Table: GSEA Results Summary

  

Fig 1: Enrichment plot: IRF5\_TARGET\_GENES      
 Profile of the Running ES Score & Positions of GeneSet Members on the Rank Ordered List

  

| SYMBOL | TITLE | RANK IN GENE LIST | RANK METRIC SCORE | RUNNING ES | CORE ENRICHMENT || 1 | 51134 | CEP83 | 19 | 1.038 | 0.0153 | Yes |
| 2 | 284942 | RPL23AP82 | 48 | 0.923 | 0.0207 | Yes |
| 3 | 23322 | RPGRIP1L | 60 | 0.871 | 0.0372 | Yes |
| 4 | 3770 | KCNJ14 | 157 | 0.721 | -0.0137 | Yes |
| 5 | 51434 | ANAPC7 | 166 | 0.710 | 0.0005 | Yes |
| 6 | 54971 | BANP | 172 | 0.707 | 0.0168 | Yes |
| 7 | 29940 | DSE | 174 | 0.703 | 0.0360 | Yes |
| 8 | 23450 | SF3B3 | 178 | 0.697 | 0.0536 | Yes |
| 9 | 11231 | SEC63 | 193 | 0.685 | 0.0626 | Yes |
| 10 | 3308 | HSPA4 | 194 | 0.685 | 0.0820 | Yes |
| 11 | 9456 | HOMER1 | 198 | 0.680 | 0.0991 | Yes |
| 12 | 3304 | HSPA1B | 207 | 0.669 | 0.1121 | Yes |
| 13 | 22948 | CCT5 | 215 | 0.663 | 0.1257 | Yes |
| 14 | 55028 | C17orf80 | 222 | 0.659 | 0.1400 | Yes |
| 15 | 55599 | RNPC3 | 227 | 0.652 | 0.1555 | Yes |
| 16 | 64794 | DDX31 | 248 | 0.633 | 0.1586 | Yes |
| 17 | 55756 | INTS9 | 257 | 0.624 | 0.1703 | Yes |
| 18 | 116143 | WDR92 | 267 | 0.619 | 0.1812 | Yes |
| 19 | 36 | ACADSB | 302 | 0.589 | 0.1726 | Yes |
| 20 | 84946 | LTV1 | 319 | 0.583 | 0.1772 | Yes |
| 21 | 6715 | SRD5A1 | 325 | 0.578 | 0.1899 | Yes |
| 22 | 51491 | NOP16 | 334 | 0.573 | 0.2002 | Yes |
| 23 | 100529261 | CHURC1-FNTB | 343 | 0.570 | 0.2105 | Yes |
| 24 | 57109 | REXO4 | 370 | 0.552 | 0.2068 | Yes |
| 25 | 5718 | PSMD12 | 372 | 0.552 | 0.2217 | Yes |
| 26 | 23567 | ZNF346 | 393 | 0.541 | 0.2222 | Yes |
| 27 | 387263 | C6orf120 | 400 | 0.535 | 0.2329 | Yes |
| 28 | 83932 | SPRTN | 404 | 0.535 | 0.2458 | Yes |
| 29 | 56894 | AGPAT3 | 414 | 0.527 | 0.2541 | Yes |
| 30 | 63929 | XPNPEP3 | 416 | 0.526 | 0.2682 | Yes |
| 31 | 148304 | C1orf74 | 447 | 0.506 | 0.2603 | No |
| 32 | 23383 | MAU2 | 459 | 0.500 | 0.2663 | No |
| 33 | 4191 | MDH2 | 494 | 0.476 | 0.2545 | No |
| 34 | 5378 | PMS1 | 506 | 0.471 | 0.2597 | No |
| 35 | 78988 | MRPL57 | 557 | 0.441 | 0.2350 | No |
| 36 | 79707 | NOL9 | 560 | 0.441 | 0.2460 | No |
| 37 | 9653 | HS2ST1 | 591 | 0.429 | 0.2359 | No |
| 38 | 79807 | GSTCD | 644 | 0.402 | 0.2086 | No |
| 39 | 11159 | RABL2A | 653 | 0.399 | 0.2140 | No |
| 40 | 11158 | RABL2B | 654 | 0.399 | 0.2253 | No |
| 41 | 4507 | MTAP | 690 | 0.384 | 0.2102 | No |
| 42 | 2932 | GSK3B | 714 | 0.367 | 0.2035 | No |
| 43 | 83743 | GRWD1 | 720 | 0.364 | 0.2101 | No |
| 44 | 55003 | PAK1IP1 | 734 | 0.359 | 0.2106 | No |
| 45 | 58509 | CACTIN | 748 | 0.351 | 0.2109 | No |
| 46 | 51154 | MRTO4 | 794 | 0.327 | 0.1867 | No |
| 47 | 9343 | EFTUD2 | 818 | 0.305 | 0.1782 | No |
| 48 | 6558 | SLC12A2 | 839 | 0.276 | 0.1712 | No |
| 49 | 3312 | HSPA8 | 848 | 0.244 | 0.1721 | No |
| 50 | 54206 | ERRFI1 | 849 | -0.229 | 0.1787 | No |
| 51 | 6520 | SLC3A2 | 878 | -0.290 | 0.1660 | No |
| 52 | 23731 | TMEM245 | 891 | -0.306 | 0.1658 | No |
| 53 | 27131 | SNX5 | 907 | -0.333 | 0.1641 | No |
| 54 | 3475 | IFRD1 | 935 | -0.360 | 0.1542 | No |
| 55 | 6397 | SEC14L1 | 941 | -0.364 | 0.1608 | No |
| 56 | 81544 | GDPD5 | 961 | -0.381 | 0.1575 | No |
| 57 | 4864 | NPC1 | 964 | -0.384 | 0.1669 | No |
| 58 | 7503 | XIST | 967 | -0.385 | 0.1764 | No |
| 59 | 3516 | RBPJ | 1021 | -0.419 | 0.1488 | No |
| 60 | 7871 | SLMAP | 1038 | -0.435 | 0.1493 | No |
| 61 | 1657 | DMXL1 | 1080 | -0.462 | 0.1319 | No |
| 62 | 5834 | PYGB | 1096 | -0.475 | 0.1342 | No |
| 63 | 117178 | SSX2IP | 1163 | -0.532 | 0.1002 | No |
| 64 | 57649 | PHF12 | 1212 | -0.588 | 0.0812 | No |
| 65 | 1983 | EIF5 | 1219 | -0.595 | 0.0936 | No |
| 66 | 440 | ASNS | 1232 | -0.609 | 0.1019 | No |
| 67 | 81573 | ANKRD13C | 1393 | -1.049 | 0.0126 | No |
Table: GSEA details [plain text format]

  

Fig 2: IRF5\_TARGET\_GENES      
 Blue-Pink O' Gram in the Space of the Analyzed GeneSet

  

Fig 3: IRF5\_TARGET\_GENES: Random ES distribution      
 Gene set null distribution of ES for **IRF5\_TARGET\_GENES**
